# Fecal DNA SDC2 methylation test for colorectal cancer diagnosis: A systematic review and meta-analysis

**DOI:** 10.17305/bb.2026.13425

**Published:** 2026-01-27

**Authors:** Xinxin Liu, Bing Yang, Dongxin Tang

**Affiliations:** 1 Department of Oncology, Pingxiang People’s Hospital, Pingxiang, China; 2 Youth League Committee, First Affiliated Hospital of Guizhou University of Traditional Chinese Medicine, Guiyang, China; 3 Department of Oncology, First Affiliated Hospital of Guizhou University of Traditional Chinese Medicine, Guiyang, China

**Keywords:** Syndecan-2, DNA methylation, stool, colorectal cancer, diagnosis

## Abstract

Fecal DNA methylation of the syndecan-2 *(SDC2)* gene is being explored as a noninvasive biomarker for colorectal cancer (CRC) detection. However, its diagnostic performance necessitates thorough evaluation. A systematic search of PubMed, Embase, and Web of Science was conducted to identify studies investigating fecal *SDC2* methylation (*mSDC2*) for CRC diagnosis. Eligible studies included adult CRC patients with histological confirmation and controls with either normal mucosa or benign colorectal lesions. Pooled sensitivity and specificity were synthesized using a Reitsma bivariate random-effects model, and summary receiver operating characteristic (SROC) curves with corresponding area under the curve (AUC) values were derived from this hierarchical model. Twenty-five studies encompassing 3,427 CRC patients, 3,267 individuals with benign lesions, and 5,372 with normal mucosa were included. For the comparison of CRC versus normal mucosa (24 studies), the pooled sensitivity and specificity were 0.86 (95% confidence interval [CI]: 0.82–0.89; I^2^ ═ 88%) and 0.93 (95% CI: 0.90–0.95; I^2^ ═ 95%), respectively. The pooled diagnostic odds ratio (DOR) was 81.73 (95% CI: 51.60–129.46), with an AUC of 0.95 (95% CI: 0.93–0.97). In the comparison against benign lesions (22 studies), the sensitivity was 0.85 (95% CI: 0.81–0.89; I^2^ ═ 87%), specificity was 0.66 (95% CI: 0.59–0.71; I^2^ ═ 91%), DOR was 11.10 (95% CI: 7.61–16.19), and AUC was 0.83 (95% CI: 0.80–0.86). Deeks’ funnel plot asymmetry tests indicated no statistically significant publication bias (*P* ═ 0.48 and 0.54). In conclusion, fecal *mSDC2* testing demonstrates high diagnostic accuracy for CRC detection when compared to individuals with normal mucosa and moderate performance against benign colorectal lesions. These findings suggest that *mSDC2* may serve as a promising noninvasive biomarker to complement existing CRC screening methodologies.

## Introduction

Colorectal cancer (CRC) is among the most prevalent malignancies worldwide and is a leading cause of cancer-related morbidity and mortality [1, 2]. The prognosis for CRC is closely associated with the stage at diagnosis, where early detection significantly enhances survival outcomes [3, 4]. While colonoscopy remains the gold standard for early detection, its invasiveness, high costs, and limited accessibility hinder compliance with population-based screening programs [5, 6]. Noninvasive alternatives, such as the fecal immunochemical test (FIT) and multi-target stool DNA (mt-sDNA) testing, have been introduced to improve participation rates [5, 6]; however, their diagnostic accuracy is suboptimal, particularly for early-stage disease and precancerous lesions [5, 6]. Consequently, there is an urgent need for accurate, noninvasive biomarkers that can reliably detect CRC at an early stage [7, 8].

The *syndecan-2* (*SDC2*) gene, a member of the syndecan family of heparan sulfate proteoglycans, plays a critical role in cell adhesion, proliferation, and migration [9, 10]. Aberrant methylation of *SDC2* (*mSDC2*) contributes to colorectal tumorigenesis by silencing tumor suppressor functions and promoting malignant transformation [10, 11]. The detection of *mSDC2* in fecal DNA—typically through quantitative methylation-specific PCR (qMSP)—represents a promising strategy for noninvasive CRC screening [12]. Fecal testing is advantageous due to its safety, convenience, and high acceptance among patients [13]. Previous meta-analyses assessing the diagnostic value of *mSDC2*, published in 2022 [14, 15], were limited by small sample sizes, inclusion of both fecal and non-fecal samples, and a lack of distinction between normal mucosa and benign colorectal lesions. Furthermore, additional studies have emerged to evaluate the role of fecal *mSDC2* in the early diagnosis of CRC [16–31]. To address these limitations, we conducted a comprehensive meta-analysis focused exclusively on fecal *mSDC2* testing for CRC diagnosis, separately summarizing its diagnostic performance against normal mucosa and benign colorectal lesions.

## Materials and methods

This systematic review and meta-analysis was performed in accordance with the Preferred Reporting Items for Systematic Reviews and Meta-Analyses (PRISMA) guidelines [32, 33] and adhered to the methodological recommendations outlined in the Cochrane Handbook [34] to ensure rigor in study design, data synthesis, and reporting. The protocol for the meta-analysis has been registered in International Prospective Register of Systematic Reviews (PROSPERO) under ID: CRD420251112337.

### Database search

To identify eligible studies, we conducted a systematic search of PubMed, Embase, and Web of Science using a combination of terms related to the biomarker (“*syndecan-2*” OR “*syndecan 2*” OR “*SDC2*”), anatomical site (“colon” OR “rectal” OR “rectum” OR “colorectal” OR “colorectum”), and disease condition (“cancer” OR “tumor” OR “neoplasms” OR “carcinoma” OR “adenocarcinoma” OR “malignancy”). No restrictions were placed on sample source or study outcomes during the search to maximize the retrieval of relevant literature; however, only studies utilizing fecal samples were eligible for inclusion according to predefined selection criteria. The search was limited to human studies published as full-text articles in English, covering the period from database inception to May 31, 2025. Additionally, we manually screened the reference lists of relevant publications to identify further eligible studies. The complete search strategies for each database are provided in Supplemental File 1.

### Study selection criteria

Studies were selected if they met the following criteria:

**Population:** Adult participants (≥18 years), including patients with histologically confirmed CRC and appropriate control groups (e.g., healthy individuals or patients with non-malignant colorectal diseases such as adenoma).

**Index test:** Studies evaluating *SDC2* gene methylation in fecal DNA samples using any valid detection method (e.g., qMSP or quantitative bisulfite next-generation sequencing [NGS]).

**Reference standard:** Diagnosis of CRC must be confirmed by histopathological examination (either via samples from endoscopic or surgical resection), which is considered the gold standard.

**Outcomes:** Studies must provide sufficient data to construct a 2×2 contingency table (i.e., true positives, false positives, false negatives, and true negatives), enabling the calculation of diagnostic performance metrics (sensitivity, specificity, etc.).

**Study design:** Original clinical studies, including cross-sectional, case-control, or cohort designs (retrospective or prospective).

**Language:** Only studies published in English were included, consistent with the journal’s scope. However, potentially relevant non-English studies may have been overlooked.

### Exclusion criteria:

(1) Studies utilizing non-fecal samples for *mSDC2* detection (e.g., blood, tissue, intestinal lavage fluid).

(2) Studies that did not report diagnostic performance specifically for CRC or failed to provide extractable 2×2 data for CRC patients.

(3) Studies that evaluated multi-gene panels but did not report the individual performance of *mSDC2*.

(4) Experimental studies involving cell lines, animal models, or *in vitro* systems rather than clinical human samples.

(5) Non-original studies, such as reviews, meta-analyses, editorials, or conference abstracts.

In cases where two studies included potentially overlapping patient populations, the study with the larger sample size was included in the meta-analysis.

### Data collection and quality assessment

Two independent reviewers screened the literature, extracted data, and evaluated study quality using predefined criteria. Discrepancies were resolved through discussion to achieve consensus. Extracted data included study characteristics (first author, publication year, country, and design), participant information (number and stage of CRC cases, control type, overall mean age, and sex distribution), details of fecal *mSDC2* testing, numbers of *mSDC2*-positive individuals in cases and controls, and the reference standard for CRC diagnosis. Study quality was assessed using the Quality Assessment Tool for Diagnostic Accuracy Studies (QUADAS-2) [35], with each study rated as having low, high, or unclear risk of bias across key domains based on risk sources and applicability.

### Statistical methods

This meta-analysis separately summarized the diagnostic performance of fecal *mSDC2* for CRC, comparing it to controls with normal colonic mucosa and those with benign colorectal lesions. Sensitivity and specificity were jointly synthesized using a Reitsma bivariate random-effects model, which accounts for the correlation between paired outcomes and between-study heterogeneity. Positive and negative diagnostic likelihood ratios and diagnostic odds ratios were derived from the pooled sensitivity and specificity estimates. The diagnostic odds ratio (DOR), indicating the odds of a correct diagnosis relative to a misdiagnosis [36], was calculated to reflect overall test accuracy. Discriminative performance was assessed using summary receiver operating characteristic (SROC) curves, with corresponding AUCs obtained from the hierarchical model rather than averaging study-level AUCs. When a study contained zero cells in the 2×2 contingency table, a continuity correction of 0.5 was applied to all four cells to enable model convergence. Summary sensitivity and specificity represent the model-implied operating point of the Reitsma bivariate random-effects model, based on thresholds reported in the original studies or, when not explicitly stated, the Youden index–optimized cutoff used by the study authors. Between-study heterogeneity was evaluated using the Cochrane *Q* test (*P* < 0.10 considered significant) [34], and quantified with the I^2^ statistic, with thresholds of <25%, 25%–75%, and >75% indicating low, moderate, and substantial heterogeneity, respectively [37]. Publication bias was examined using Deeks’ funnel plot and asymmetry test [38]. All statistical analyses were performed using STATA software (Version 17.0; Stata Corporation, College Station, TX, USA), with *P* < 0.05 regarded as statistically significant.

## Results

### Results of literature search

**Figure 1.**
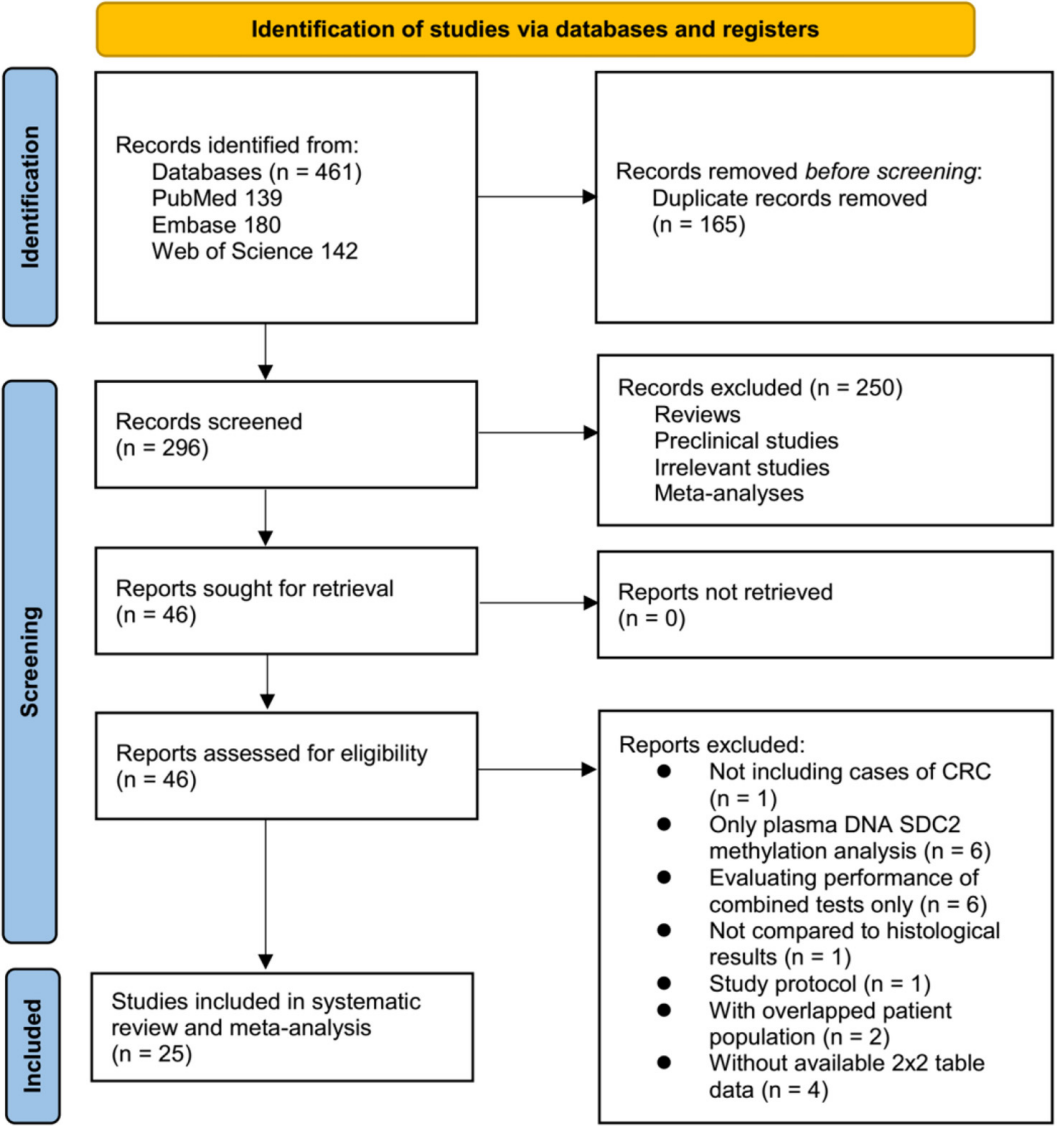
Flowchart illustrating the study screening and identification process.

The initial database search identified 461 studies, as illustrated in Figure 1. After removing 165 duplicates, 296 studies remained. Subsequent analysis of titles and abstracts led to the exclusion of an additional 250 studies that lacked relevance to the objectives of this meta-analysis, resulting in 46 studies selected for full-text review. Upon thorough evaluation of these full texts, 21 studies were excluded for reasons outlined in Figure 1. Ultimately, 25 studies [16–31], [39–47] were included in the meta-analysis.

### Study characteristics and quality assessment

The principal characteristics of the 25 studies included in this meta-analysis are summarized in Table 1. These studies were conducted in China (including Taiwan), Korea, and Thailand, and were published between 2017 and 2025. Most studies (20 out of 25) employed a prospective design [16], [18], [20–25], [27–30], [39], [40], [42–47], while five utilized a retrospective design [17, 19, 26, 31, 41]. Data from a total of 3,427 patients with CRC were included, with disease stages ranging from 0 to IV. Control groups consisted of individuals with normal mucosa, benign colorectal lesions (such as adenomas or hyperplastic polyps), or both. Overall, the meta-analysis included 5,372 participants with normal mucosa and 3,267 participants with benign colorectal lesions. The mean age of participants varied from 52.4–67.8 years, with the proportion of male participants ranging from 39.4% to 68.1%. In 24 studies, fecal *mSDC2* levels were evaluated using qMSP [16–23], [25–31], [39–47], while one study [24] employed quantitative bisulfite NGS. Cutoff thresholds were primarily determined using receiver operating characteristic curve analysis or predefined cycle-threshold values; however, some studies did not report the methods for generating these thresholds [17–19], [24], [26–28], [30], [31], [45] or the specific threshold values [20], [24–26], [28], [39], [47]. Additionally, the methods for threshold determination and precise cutoff values varied, limiting further stratified analyses by threshold definition. The reference standard for CRC diagnosis was histological confirmation via endoscopic or surgical biopsy across all studies.

**Table 1:** Characteristics of the included studies

**Study**	**Country**	**Design**	**CRC stage**	**No. of patients with CRC**	**Control characteristics**	**No. of subjects with benign colorectal lesions**	**No. of subjects with normal colonic mucosa**	**Mean age (years)**	**Men (%)**	**Methods for fecal mSDC2 evaluation**	**Methods for determination of cutoff of mSDC2**	**Cutoff values of mSDC2**	**No. of fecal mSDC2 (+) in CRC patients**	**No. of fecal mSDC2 (+) controls with benign lesions**	**No. of fecal mSDC2 (+) controls with normal mucosa**	**Reference test**
Oh 2017	Korea	P	Stages I–IV	50	Both benign lesions (adenomatous polyps) and healthy mucosa	21	22	60.6	60.2	qMSP	ROC curve analysis	Ct ≤ 40	45	7	2	Endoscopic or surgical histology
Niu 2017	China	P	Stages I–IV	196	Both benign lesions (adenomas ≥1 cm) and healthy mucosa	122	179	59.3	54.4	qMSP	ROC curve analysis	NR	159	71	12	Endoscopic histology
Han 2019	Korea	R	Stages 0–IV	245	Both benign lesions (advanced adenomas ≥1 cm, non-advanced adenomas <1 cm, hyperplastic/other polyps) and healthy mucosa	44	245	60.5	52.5	qMSP	Pre-specified based on prior pilot study results	Ct ≤ 40	221	12	24	Endoscopic or surgical histology
Sun 2019	China	P	Stages I–IV	105	Both benign (adenoma; hyperplastic polyps) and healthy mucosa	26	102	NR	53	qMSP	Assay’s analytical detection limit	Ct ≤ 42	72	10	9	Endoscopic histology
Wang 2020	China	P	Stages I–IV	359	Both benign (advanced adenoma) and healthy mucosa	38	512	54.1	51.3	qMSP	Cutoff selected to maximize sensitivity and minimize the false positive rate	Ct ≤ 38	301	16	14	Endoscopic histology
Su 2021	Taiwan (China)	P	Stages 0–IV	62	Healthy mucosa only	0	76	58.3	50.5	qMSP	Cutoffs selected to balance overall sensitivity and specificity	Ct ≤ 39	48	0	9	Endoscopic histology
Zhang 2021	China	P	Stages I–IV	61	Both benign (adenoma) and healthy mucosa	16	53	54.5	58.5	qMSP	NR	Ct ≤ 38	47	11	1	Endoscopic or surgical histology
Dai 2022	China	P	Stages 0–IV	244	Benign (advanced adenomas; small polyp) and healthy mucosa	98	187	55.6	52.9	qMSP	ROC curve analysis	Ct ≤ 40	204	42	9	Endoscopic or surgical histology
Ma 2022	China	P	Stages 0–IV	102	Benign (advanced adenoma; non-advanced adenomas; colitis) and healthy mucosa	107	130	61	49	qMSP	ROC curve analysis	NR	89	31	7	Endoscopic or surgical histology
Li 2023a	China	P	Stages I–IV	30	Healthy mucosa only	0	30	52.4	51.7	qMSP	ROC curve analysis	Ct ≤ 38	28	0	3	Endoscopic or surgical histology
Liu 2023	China	R	Stages I–IV	263	Both benign (adenoma/polyps) and healthy mucosa	512	445	NR	61.2	qMSP	NR	Ct ≤ 38	220	195	140	Endoscopic or surgical histology
Xu 2023	China	P	Stages I–IV	42	Benign (advanced adenoma) and healthy mucosa	302	1345	60	48.1	qMSP	NR	Ct ≤ 38.5	39	106	94	Endoscopic histology
Cheng 2023	China	P	Stages I–III	50	Benign (advanced adenoma) and healthy mucosa	50	50	58.9	57.3	qMSP	ROC curve analysis	Ct ≤ 38	49	18	25	Endoscopic histology
Zeng 2023	China	R	Stages 0–IV	150	Benign (advanced adenoma) and healthy mucosa	23	275	NR	68	qMSP	NR	Ct ≤ 38	119	7	21	Endoscopic histology
Li 2023b	China	P	Stages 0–IV	105	Both benign (adenoma/polyps) and healthy mucosa	158	100	54.3	59.2	qMSP	ROC curve analysis	Ct ≤ 38	86	52	1	Endoscopic histology
Zhan 2023	China	P	Stages 0–IV	445	Both benign lesions (adenomas, non-neoplastic GI diseases) and healthy mucosa	472	62	NR	62.9	qMSP	ROC curve analysis	NR	310	78	7	Endoscopic histology
Kim 2024	Korea	P	Stages 0–IV	20	Both benign lesions (advanced adenomas) and healthy mucosa	73	384	67.8	49.6	qMSP	ROC curve analysis	Ct ≤ 40	19	35	71	Endoscopic histology
Lohsiriwat 2024	Thailand	P	NR	47	Both benign lesions (advanced adenomas, non-advanced adenomas) and healthy mucosa	60	150	62.1	39.4	qMSP	ROC curve analysis	NR	43	11	11	Endoscopic histology
Liu 2024	China	P	Stages I–IV	83	Healthy mucosa only	0	98	60	65.1	quantitative bisulfite NGS	NR	NR	76	0	0	Endoscopic or surgical histology
Long 2024	China	R	Stages 0–IV	138	Both benign (advanced adenoma/polyps) and healthy mucosa	62	28	57.8	53.2	qMSP	NR	NR	102	13	2	Endoscopic histology
Zhang 2024	China	P	Stages I–IV	403	Both benign (advanced or non-advanced adenomas) and healthy mucosa	219	210	60.9	61.6	qMSP	NR	NR	371	82	12	Endoscopic histology
Zhao 2024	China	P	Stages I–IV	26	Only benign lesions (adenoma, hyperplastic polyps, etc.)	382	0	NR	40.6	qMSP	ROC curve analysis	Ct ≤ 39	22	118	0	Endoscopic histology
Zou 2024	China	P	Stages I–IV	116	Benign (adenoma) and healthy mucosa	31	44	59.8	57.6	qMSP	NR	Ct ≤ 38	85	12	1	Endoscopic histology
Luo 2024	China	P	Stages 0–IV	16	Benign lesions (adenoma, hyperplastic polyps, etc.) and healthy mucosa	404	615	52	48.5	qMSP	NR	Ct ≤ 38	14	46	27	Endoscopic histology
Liu 2025	China	R	Stages I–IV	69	Benign (adenoma) and healthy mucosa	47	30	64	68.1	qMSP	NR	Ct ≤ 38	68	28	2	Endoscopic histology

Abbreviations: CRC: Colorectal cancer; CT: Cycle threshold; NR: Not reported; P: Prospective; R: Retrospective; qMSP: Quantitative methylation-specific PCR; NGS: Next-generation sequencing; mSDC2: Methylated syndecan-2.

**Table 2:** Evaluation of study quality using the QUADAS-2 scale

	**Risk of bias**				**Applicability concerns**		
**Study**	**Patient selection**	**Index test**	**Reference standard**	**Flow and timing**	**Patient selection**	**Index test**	**Reference standard**
Oh 2017	Low risk	Low risk	Low risk	Low risk	Low risk	Low risk	Low risk
Niu 2017	Low risk	Low risk	Low risk	Unclear	Low risk	Low risk	Low risk
Han 2019	High risk	Low risk	Low risk	Low risk	Low risk	Low risk	Low risk
Sun 2019	Low risk	Low risk	Low risk	Low risk	Low risk	Low risk	Low risk
Wang 2020	Low risk	Low risk	Low risk	Low risk	Low risk	Low risk	Low risk
Su 2021	Low risk	Low risk	Low risk	Unclear	Low risk	Low risk	Low risk
Zhang 2021	Low risk	Low risk	Low risk	Low risk	Low risk	Low risk	Low risk
Dai 2022	Low risk	Low risk	Low risk	Low risk	Low risk	Low risk	Low risk
Ma 2022	Low risk	Low risk	Low risk	Low risk	Low risk	Low risk	Low risk
Li 2023a	Low risk	Low risk	Low risk	Unclear	Low risk	Low risk	Low risk
Liu 2023	High risk	Low risk	Low risk	Unclear	Low risk	Low risk	Low risk
Xu 2023	Low risk	Low risk	Low risk	Unclear	Low risk	Low risk	Low risk
Cheng 2023	Low risk	Low risk	Low risk	Low risk	Low risk	Low risk	Low risk
Zeng 2023	High risk	Low risk	Low risk	Low risk	Low risk	Low risk	Low risk
Li 2023b	Low risk	Low risk	Low risk	Low risk	Low risk	Low risk	Low risk
Zhan 2023	Low risk	Low risk	Low risk	Low risk	Low risk	Low risk	Low risk
Kim 2024	Low risk	Low risk	Low risk	Low risk	Low risk	Low risk	Low risk
Lohsiriwat 2024	Low risk	Low risk	Low risk	Low risk	Low risk	Low risk	Low risk
Liu 2024	Low risk	Low risk	Low risk	Unclear	Low risk	Low risk	Low risk
Long 2024	High risk	Low risk	Low risk	Low risk	Low risk	Low risk	Low risk
Zhang 2024	Low risk	Low risk	Low risk	Low risk	Low risk	Low risk	Low risk
Zhao 2024	Low risk	Low risk	Low risk	Unclear	Low risk	Low risk	Low risk
Zou 2024	Low risk	Low risk	Low risk	Low risk	Low risk	Low risk	Low risk
Luo 2024	Low risk	Low risk	Low risk	Low risk	Low risk	Low risk	Low risk
Liu 2025	High risk	Low risk	Low risk	Unclear	Low risk	Low risk	Low risk

Abbreviation: QUADAS-2: Quality Assessment of Diagnostic Accuracy Studies 2.

Quality assessment was conducted using the QUADAS-2 tool, with results detailed in Table 2. Most studies were rated as having a low risk of bias across all domains. However, five studies [17, 19, 26, 31, 41] were deemed to have a high risk of bias in the domain of patient selection due to their retrospective design or unclear sampling methods. Furthermore, the domain of flow and timing was rated as unclear in eight studies [17, 18, 21, 24, 29, 31, 39, 44], primarily due to insufficient reporting on the interval between index testing and confirmation by the reference standard. All other domains, including those related to applicability concerns, were rated as low risk across all studies.

### Performance of fecal mSDC2 in detecting CRC vs normal mucosa

**Figure 2.**
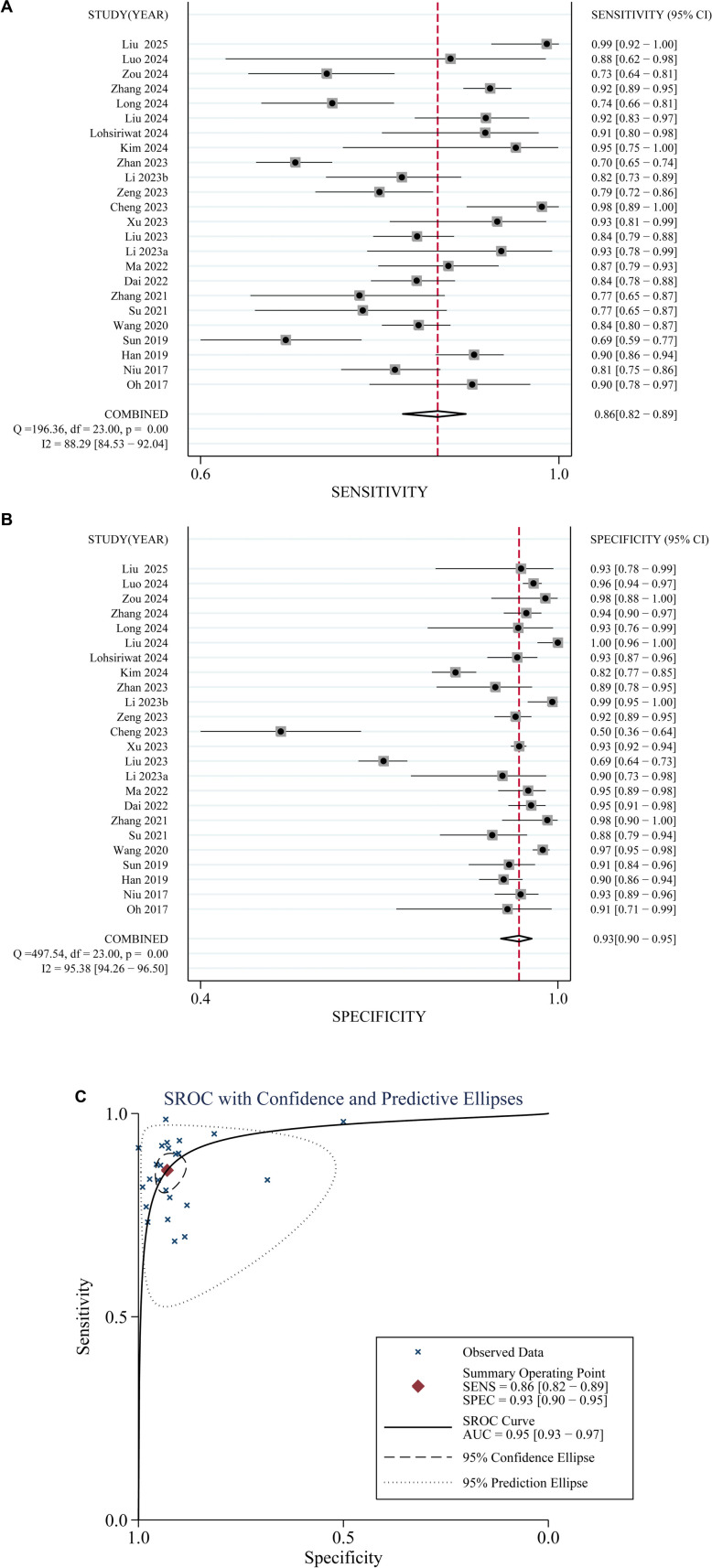
**Forest plots and summarized ROC curve illustrating the diagnostic performance of fecal *mSDC2* for detecting CRC versus normal colonic mucosa across 24 studies.** (A) Forest plot of pooled sensitivity: 0.86 (95% CI: 0.82–0.89; I^2^ ═ 88%). (B) Forest plot of pooled specificity: 0.93 (95% CI: 0.90–0.95; I^2^ ═ 95%). (C) Summarized ROC curve with AUC = 0.95 (95% CI: 0.93–0.97). The x-axis for the summarized ROC curve is presented with specificity on a reversed scale (1.0 → 0.0), corresponding to plotting 1-specificity (false-positive rate) on a forward scale. Abbreviation: CRC: Colorectal cancer.

Pooled data from 24 studies [16–28], [30], [31], [39–47] demonstrated that fecal *mSDC2* exhibited robust diagnostic performance in distinguishing CRC from individuals with normal colonic mucosa. The combined sensitivity and specificity were 0.86 (95% confidence interval (CI): 0.82–0.89; I^2^ ═ 88%, Figure 2A) and 0.93 (95% CI: 0.90–0.95; I^2^ ═ 95%, Figure 2B), respectively. The pooled positive and negative diagnostic likelihood ratios were 12.28 (95% CI: 8.39–18.01) and 0.15 (95% CI: 0.11–0.19), respectively, resulting in a DOR of 81.73 (95% CI: 51.60–129.46). The area under the summary receiver operating characteristic curve (AUC) was 0.95 (95% CI: 0.93–0.97; Figure 2C), indicating excellent diagnostic accuracy.

### Performance of fecal mSDC2 in detecting CRC vs benign lesions

**Figure 3.**
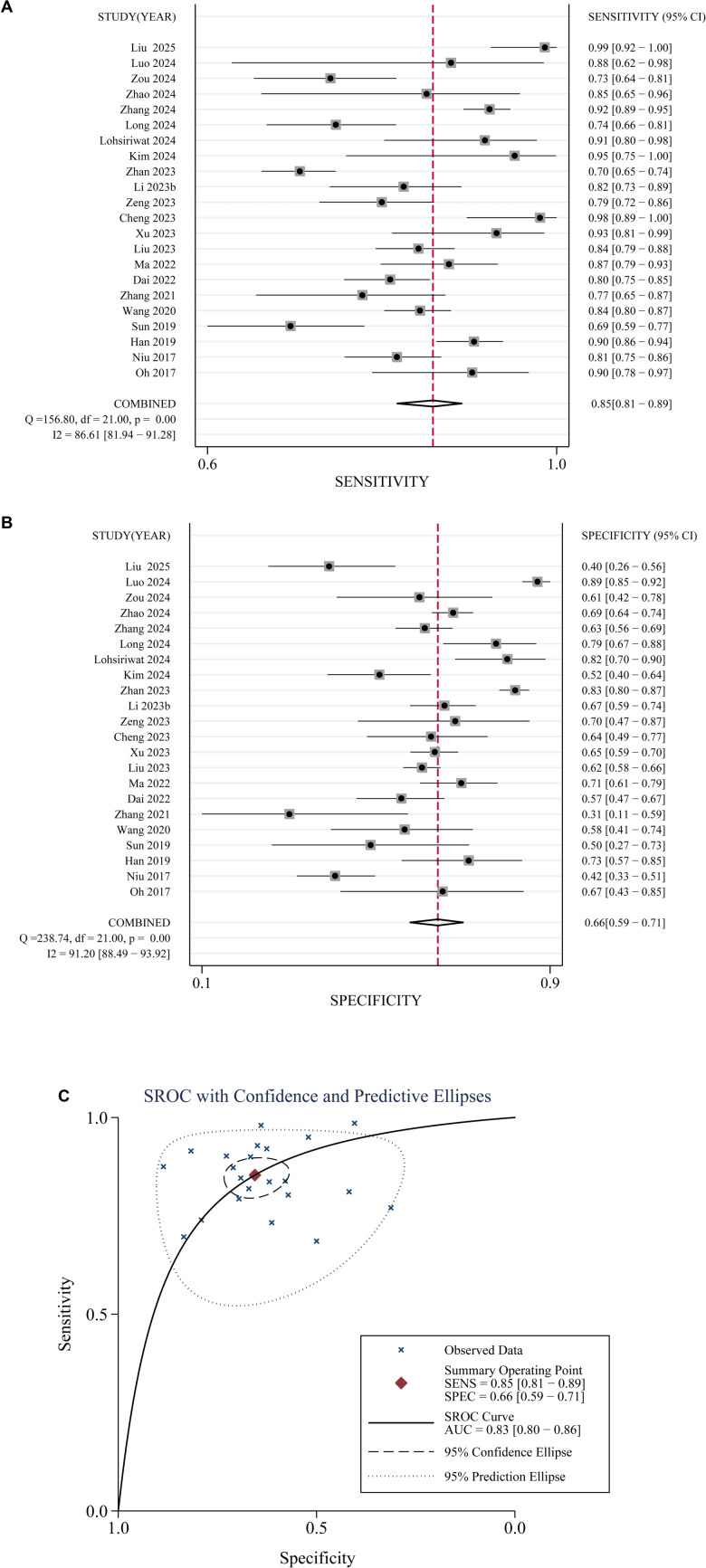
**Forest plots and summarized ROC curve illustrating the diagnostic performance of fecal *mSDC2* in distinguishing CRC from benign colorectal lesions across 22 studies.** (A) Forest plot of pooled sensitivity: 0.85 (95% CI: 0.81–0.89; I^2^ ═ 87%). (B) Forest plot of pooled specificity: 0.66 (95% CI: 0.59–0.71; I^2^ ═ 91%). (C) Summarized ROC curve with AUC = 0.83 (95% CI: 0.80–0.86). The x-axis for the summarized ROC curve is presented with specificity on a reversed scale (1.0 → 0.0), corresponding to plotting 1-specificity (false-positive rate) on a forward scale. Abbreviation: CRC: Colorectal cancer.

Pooled results from 22 studies [16–20], [22], [23], [25–31], [39–43], [45–47] revealed that fecal *mSDC2* exhibited acceptable diagnostic performance in differentiating CRC from benign colorectal lesions. The combined sensitivity was 0.85 (95% CI: 0.81–0.89; I^2^= 87%, Figure 3A), while specificity was 0.66 (95% CI: 0.59–0.71; I^2^ ═ 91%, Figure 3B). The pooled positive and negative diagnostic likelihood ratios were 2.48 (95% CI: 2.08–2.96) and 0.22 (95% CI: 0.17–0.29), respectively, yielding a DOR of 11.10 (95% CI: 7.61–16.19). The AUC was 0.83 (95% CI: 0.80–0.86; Figure 3C), indicating a moderate diagnostic accuracy of fecal *mSDC2* in distinguishing CRC from benign colorectal lesions.

### Publication bias

**Figure 4.**
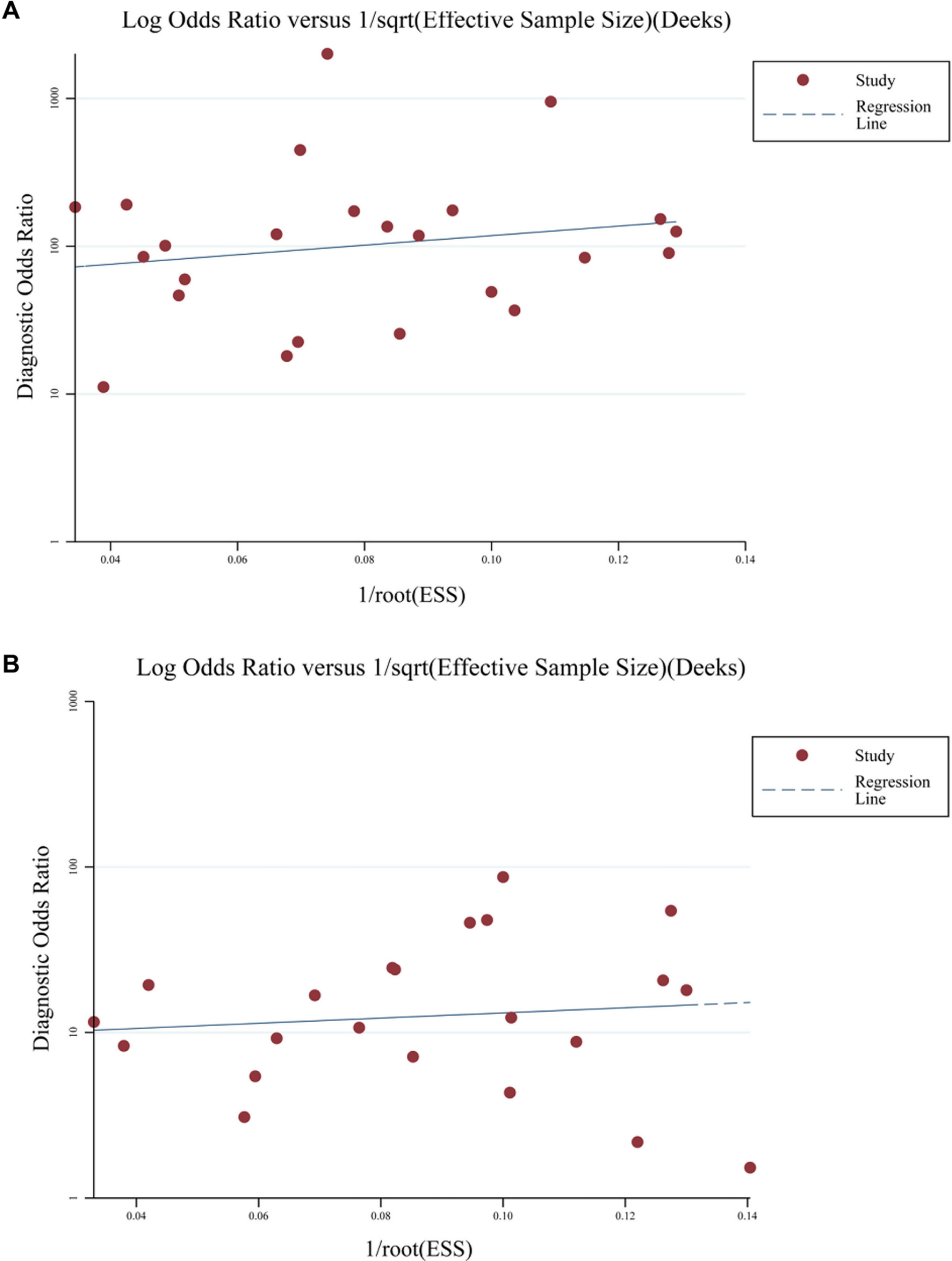
**Deeks’ funnel plots assessing potential publication bias in the diagnostic accuracy meta-analyses of fecal *mSDC2*.** (A) Deeks’ funnel plot for studies comparing CRC versus normal colonic mucosa (*P* ═ 0.48). (B) Deeks’ funnel plot for studies comparing CRC versus benign colorectal lesions (*P* ═ 0.54). Abbreviation: CRC: Colorectal cancer.

Deeks’ funnel plots for the meta-analyses summarizing the performance of fecal *mSDC2* in detecting CRC vs normal mucosa and benign colorectal lesions are presented in Figure 4A and 4B. These plots did not indicate statistically significant publication bias (*P* ═ 0.48 and 0.54). However, these findings should be interpreted cautiously, given the limited power of the test in diagnostic accuracy meta-analyses.

## Discussion

This meta-analysis comprehensively evaluated the diagnostic performance of fecal *mSDC2* testing for CRC by synthesizing data from 25 studies, including over 12,000 participants. Our findings suggest that fecal *mSDC2* demonstrates excellent diagnostic accuracy for distinguishing CRC from individuals with normal colonic mucosa and moderate performance in differentiating CRC from benign colorectal lesions. By separately analyzing these two clinically relevant comparison groups, this study provides nuanced insights into the utility of fecal *mSDC2* testing for early screening and triaging strategies in practice.

The mechanistic role of *mSDC2* in CRC detection is well-supported. The *SDC2* gene encodes a transmembrane heparan sulfate proteoglycan involved in critical biological processes, including cell proliferation, adhesion, and migration [48, 49]. Aberrant methylation of the *SDC2* promoter region results in transcriptional silencing, disrupting normal epithelial cell behavior and promoting colorectal carcinogenesis [50, 51]. *SDC2* hypermethylation has been documented in both early-stage CRC and advanced adenomas, establishing its potential as a promising biomarker for early detection [11]. Notably, tumor-derived DNA is shed into the intestinal lumen and eventually expelled in feces, where methylated gene targets such as *mSDC2* can be captured and amplified using qMSP or similar technologies [52, 53]. The noninvasive nature of stool-based collection, coupled with the high specificity of methylation detection, underscores the clinical relevance of *mSDC2* as a biomarker for CRC screening [54].

The current analysis presents several clinically significant observations. First, fecal mSDC2 demonstrates excellent diagnostic efficacy compared to normal mucosa, with pooled sensitivity and specificity exceeding 85% and 90%, respectively, and an AUC of 0.95. This performance is favorable when compared to widely used noninvasive tests, such as FIT [55] and mt-sDNA [56]. Second, when assessing benign lesions, including non-neoplastic polyps and inflammatory conditions, mSDC2 shows moderate specificity. This finding reflects the biological continuum between benign and malignant lesions, indicating that mSDC2 may be less effective in differentiating low-risk lesions from early-stage malignancies [57]. Nevertheless, its strong sensitivity in both comparisons underscores the utility of mSDC2 testing in initial screening settings, particularly for prioritizing individuals for further diagnostic evaluations, such as colonoscopy. Clinically, the high pooled sensitivity observed in this meta-analysis suggests that fecal mSDC2 testing could significantly enhance the post-test probability of CRC detection in screening contexts, especially in populations with a notable baseline risk. However, the exact magnitude of post-test risk reduction is contingent upon underlying disease prevalence, which varies across screening and clinical environments. Furthermore, while several included studies reported favorable detection rates in early-stage CRC, inconsistent reporting of stage-specific diagnostic data precluded pooled analyses by cancer stage. Future research should provide stage-stratified accuracy estimates to better delineate the role of fecal mSDC2 in early detection and precancerous lesion interception.

Moderate to substantial heterogeneity was observed, a common characteristic of meta-analyses assessing diagnostic test accuracy. In this study, heterogeneity primarily arises from variations in assay thresholds, cutoff-determination strategies, and control group composition (normal mucosa vs benign lesions), rather than study design or overall quality. Due to the variable reporting and lack of standardization in cutoff definitions, formal threshold-based subgroup or meta-regression analyses were not methodologically reliable. Additionally, sensitivity analyses excluding retrospective or high-risk-of-bias studies were not conducted, as diagnostic accuracy estimates are largely influenced by index test performance and reference standards rather than temporal study design; such exclusions would significantly reduce sample size without adequately addressing the principal sources of heterogeneity. Importantly, the use of a hierarchical bivariate random-effects model allows for robust estimation of pooled sensitivity, specificity, and AUC while appropriately accounting for between-study variability. Notably, several studies reported relatively higher *mSDC2* positivity among normal-mucosa controls—most prominently in Cheng et al. 2023 [16] and Liu et al. 2023 [17], where positivity exceeded 30%–50%, with lesser extent in Kim et al. 2024 [23] and Zhan et al. 2023 [20]. These findings likely contributed to between-study heterogeneity and attenuation of pooled specificity estimates, potentially reflecting differences in population risk profiles, assay thresholds, or background epigenetic alterations rather than true diagnostic failure.

This meta-analysis possesses several notable strengths. It represents the most current and comprehensive synthesis of fecal *mSDC2* testing for CRC diagnosis, encompassing 25 studies with a relatively large sample size. Unlike previous meta-analyses, we limited inclusion to studies using fecal samples, excluded studies utilizing tissue or plasma, and separately summarized diagnostic performance based on the type of control group—either benign lesions or normal mucosa. This approach facilitates a more clinically relevant interpretation. Furthermore, publication bias was not evident in either analysis, indicating the stability of our pooled results. However, several limitations should be acknowledged. First, although most included studies were prospective, five were retrospective, which may introduce selection or recall bias [58]. Second, the cutoff thresholds and platforms used for *mSDC2* detection varied across studies, potentially contributing to the moderate-to-high heterogeneity in sensitivity and specificity estimates. Future studies are necessary to identify optimal cutoff thresholds for fecal *mSDC2* testing in CRC diagnosis. Third, the stage distribution of CRC was inconsistently reported, preventing subgroup analysis of diagnostic accuracy by cancer stage. Additionally, while benign colorectal lesions represent a heterogeneous group, most included studies did not report diagnostic outcomes stratified by advanced vs non-advanced lesions, hindering separate analyses for high-risk precancerous neoplasia. Fourth, although we stratified analyses by control type, other patient-level factors such as age, sex, family history, or comorbidities could not be accounted for due to the lack of individual participant data. Moreover, the restriction to English-language publications may have introduced language bias, particularly given the substantial body of *SDC2* research from East Asia. Nonetheless, most large, high-quality diagnostic studies in this field are available in English. Additionally, all included studies were conducted in Asian populations, reflecting the current geographic focus of fecal *mSDC2* research. While Deeks’ funnel plot did not indicate significant asymmetry, publication bias assessment remains qualitative and exploratory, necessitating further validation of findings in non-Asian populations. Lastly, while fecal *mSDC2* shows promise, formal head-to-head diagnostic accuracy comparisons with established screening tools (e.g., FIT and mt-sDNA) remain limited. However, comparative evidence with FIT is emerging from real-world/community screening studies where both tests were applied within the same screening context, providing preliminary context for their relative performance [53]; further standardized, prospective head-to-head evaluations are warranted.

Despite these limitations, the findings of this study have relevant clinical implications. Fecal *mSDC2* testing may serve as a complementary tool to existing CRC screening modalities, particularly for individuals at average risk or those unwilling or unable to undergo colonoscopy. Its high specificity and sensitivity against normal mucosa make it attractive for initial screening in asymptomatic populations, while moderate performance against benign lesions suggests it may also aid in risk stratification among patients with detected polyps. From a public health perspective, implementing such a noninvasive and cost-effective tool could enhance screening uptake and reduce the burden of CRC-related mortality through earlier detection [59].

Future research should *focus on standardizing mSDC2* detection protocols, including optimal methylation thresholds and target sequences, to improve comparability across studies. Additionally, large-scale prospective screening trials are warranted to assess the performance of fecal *mSDC2* in average-risk populations and evaluate its additive value when combined with other noninvasive tests. Studies concentrating on longitudinal monitoring of methylation markers may also provide insights into the utility of *mSDC2* for surveillance in high-risk groups or post-polypectomy follow-up. Finally, economic evaluations are needed to establish the cost-effectiveness and feasibility of integrating *mSDC2* testing into routine screening programs.

## Conclusion

In conclusion, this meta-analysis indicates that fecal *mSDC2* testing exhibits good diagnostic performance for detecting CRC, particularly when compared to individuals with normal mucosa, and demonstrates moderate discriminative ability in distinguishing CRC from benign colorectal lesions. These findings suggest that fecal *mSDC2* may serve as a promising noninvasive biomarker to complement existing CRC screening strategies. However, given the substantial heterogeneity, limited comparative data with established screening tests, and the predominance of evidence from Asian populations, further large-scale, prospective, and geographically diverse studies—including stage-specific and head-to-head evaluations—are warranted to better define its clinical role.

## Supplemental data

**Supplemental file 1.** Detailed search strategy for each database

**PubMed**

(“syndecan-2”[Mesh] OR “syndecan-2”[tiab] OR “syndecan 2”[tiab] OR “SDC2”[tiab]) AND (“colorectal neoplasms”[Mesh] OR “colorectal”[tiab] OR “colorectum”[tiab] OR “colon”[tiab] OR “rectal”[tiab] OR “rectum”[tiab]) AND (“neoplasms”[Mesh] OR “carcinoma”[tiab] OR “cancer”[tiab] OR “tumor”[tiab] OR “malignancy”[tiab] OR “adenocarcinoma”[tiab])

Filters: Humans, English, Publication date from inception to 2025/05/31

**Embase**

(‘syndecan 2’/exp OR ‘syndecan-2’:ti,ab OR ‘syndecan 2’:ti,ab OR sdc2:ti,ab) AND (‘colorectal tumor’/exp OR colorectal:ti,ab OR colorectum:ti,ab OR colon:ti,ab OR rectal:ti,ab OR rectum:ti,ab) AND (‘neoplasm’/exp OR carcinoma:ti,ab OR cancer:ti,ab OR tumor:ti,ab OR malignancy:ti,ab OR adenocarcinoma:ti,ab)

Limits: Human, English, Publication date from inception to 2025/05/31

**Web of Science**

TS=(“syndecan-2” OR “syndecan 2” OR “SDC2”) AND TS=(“colorectal” OR “colorectum” OR “colon” OR “rectal” OR “rectum”) AND TS=(“neoplasms” OR “carcinoma” OR “cancer” OR “tumor” OR “malignancy” OR “adenocarcinoma”)

Refined by:

Languages: (English)

Document Types: (Article)

Timespan: All years – 2025-05-31

## Data Availability

All data generated or analyzed during this study are included in this published article.
